# Supplementary material for: DNA methylation modules associate with incident cardiovascular disease and cumulative risk factor exposure
Source: Clin Epigenetics. 2019 Oct 15;11:142. doi: 10.1186/s13148-019-0705-2 (PMC6792327; doi:10.1186/s13148-019-0705-2)
Supplement: Supplementary file 1 — Supplementary Information (PDF 153 kb) [file 13148_2019_705_MOESM1_ESM.pdf]

Supplementary Info: DNA methylation modules associate with incident cardiovascular disease and cumulative risk factor exposure

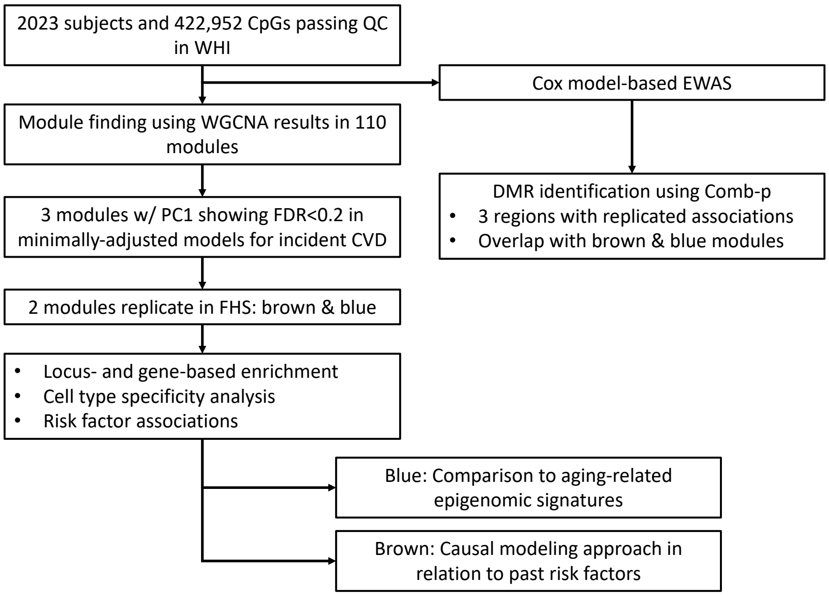

Figure S1: Study overview, including module- and region-based analyses as well as follow-up.

Table S1: P-values for module associations with incident CVD in discovery and replication.

	WHI (discovery)		FHS (replication)		
Module	Partially adjusted	Fully adjusted	Partially adj.	Partially adj. (mixed)	Fully adjusted
blue	0.0002736	0.0500018	0.0000085	0.0000085	0.8189348
brown4	0.0045462	0.0872688	0.0000962	0.0000963	0.0997390
lavenderblush3	0.0050028	0.0210976	0.0202819	0.0202804	0.1580861

[1] Partially-adjusted models are adjusted for technical covariates (DNA pull batch in WHI and study center + 7 control probe PCs in FHS) and estimated cell counts. Fully-adjusted models are additionally adjusted for age, sex, smoking status and smoking pack-years.
[2] Mixed model contains a random intercept for each family.

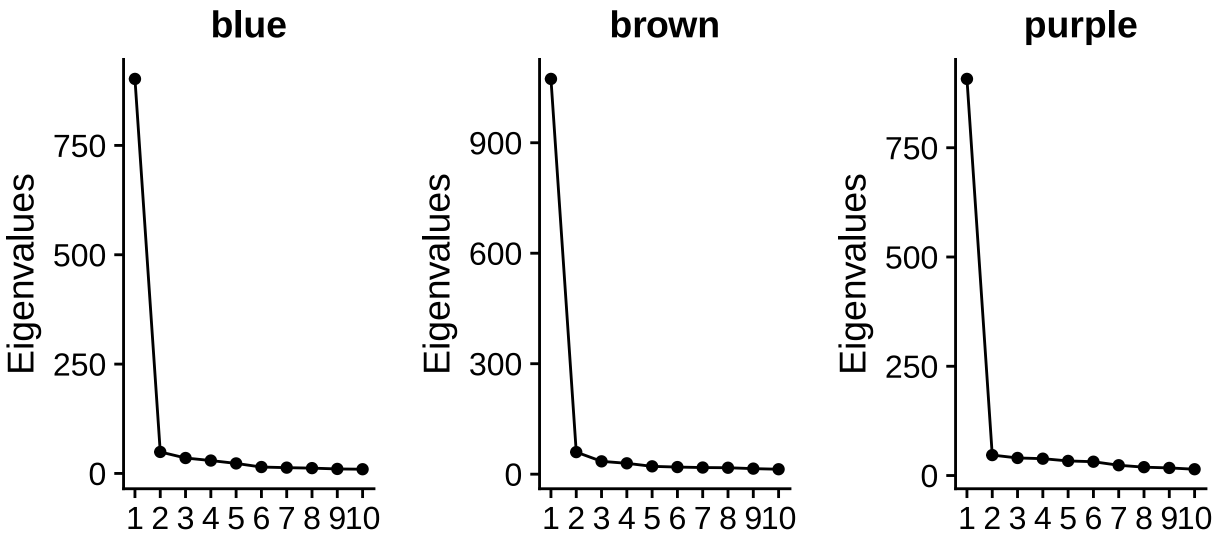

Figure S2: Scree plots for PCA on the set of CpGs corresponding to each of the top modules.

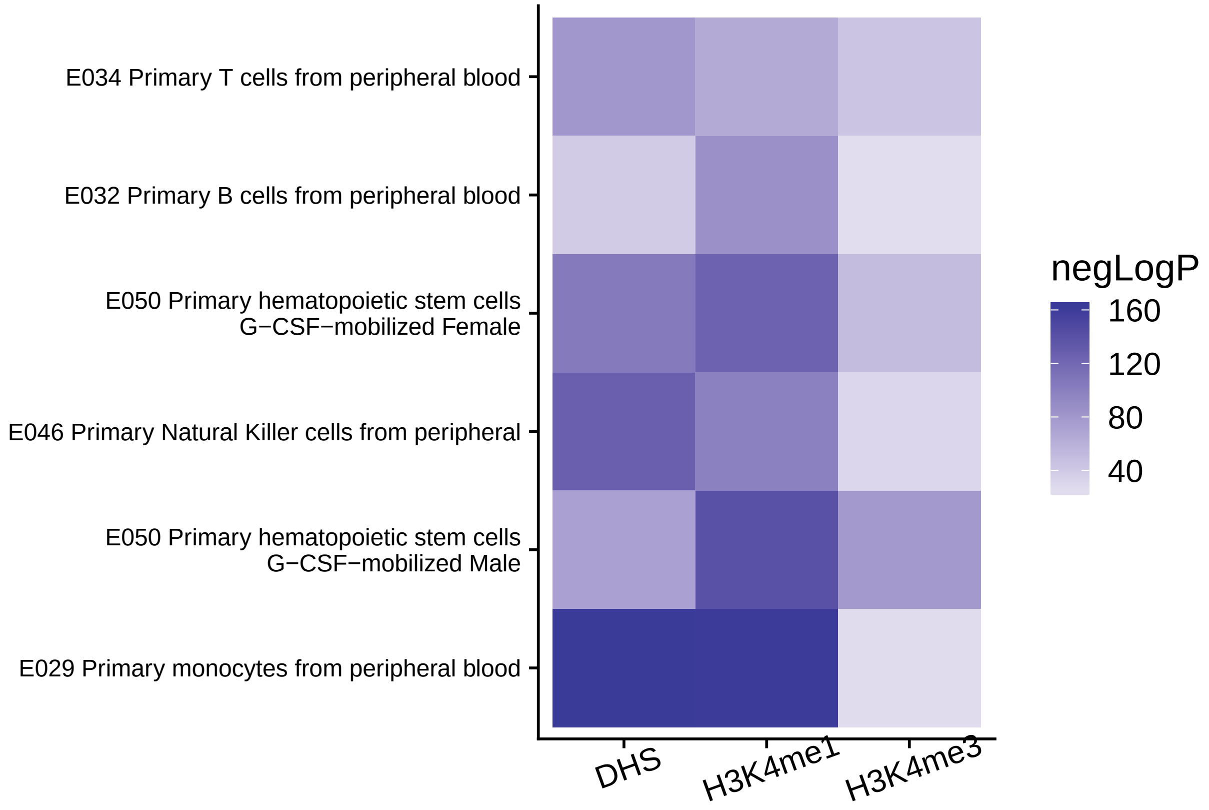

Figure S3: eFORGE cell type-specificity plot for the brown module.

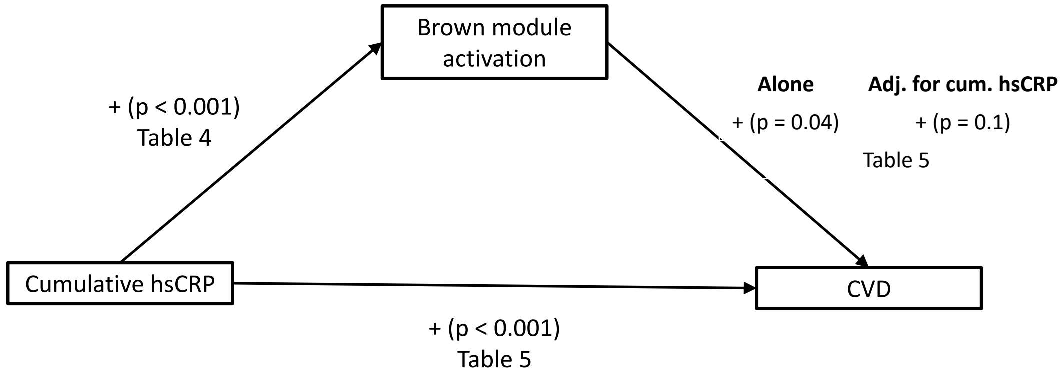

Figure S4: Example diagram of cumulative risk factor mediation by brown methylation module activation. Results from 4 regressions are shown: cumulative risk factor exposure to brown activation, cumulative risk factor exposure to incident CVD, and brown activation to incident CVD with and without adjustment for cumulative risk factor exposure. Regression terms represented as: sign of coefficient (p-value).

Table S2: CpGs with FDR < 0.05 in the discovery set (Bonferroni threshold = 1.18e-7)

CpG	Chromosome	Dir. of Assoc.	P-value	Location	Annotated Gene	Replication P-value
cg09155044	chr16	+	6.63e-09	TSS1500	VKORC1	0.107
cg24434800	chr1	+	5.04e-08			0.629
cg11691298	chr2	+	1.1e-07	Body	FAM59B	0.525
cg02379107	chr20	+	4.72e-07	TSS1500	KIAA1755	0.930
